# A pilot study investigating immediate effects of vergence training on binocular function and reaction time in youth badminton athletes

**DOI:** 10.1038/s41598-025-28743-7

**Published:** 2025-12-04

**Authors:** Yuh-Ling Shyu, Ching-Wen Huang, Shou-Chun Wei, Shang -Min Yeh, Chien-Te Liu, Shuan-Yu Huang, Yun-Wei Chiang

**Affiliations:** 1 Department of Athletics, Central Taiwan University of Science and Technology, Taichung, 406 Taiwan; 2 Department of Ophthalmology, Fu Jen Catholic University Hospital, New Taipei City, 242 Taiwan; 3 Department of Optometry, Central Taiwan University of Science and Technology, Taichung, 406 Taiwan; 4 School of Medicine, Chung Shan Medical University, Taichung, 402 Taiwan

**Keywords:** Vergence training, Automatic dual rotational risley prisms (ADRRPs), Binocular function, Reaction time, Badminton athletes, Health care, Medical research, Neuroscience, Psychology, Psychology

## Abstract

This pilot study examined the immediate effects of a single session of vergence training using automatic dual-rotational Risley prisms (ADRRPs) on binocular visual function and reaction time in youth badminton athletes. Twenty-six participants were randomly assigned to a visual training (VT) group (*n* = 16) or a control group (*n* = 10). After a 15-minute ADRRPs session, the VT group showed greater short-term improvements in vergence facility (VF) and accommodative facility (AF) than the control group, and a reduction in reaction time was also observed. Other parameters, including amplitude of accommodation (AA), near point of convergence (NPC), and positive fusional vergence (PFV), showed minimal or non-significant changes, suggesting limited short-term oculomotor adaptation. These findings indicate that brief, automated vergence interventions may be feasible for enhancing specific visual functions and visuomotor responsiveness in athletes. However, given the small and uneven sample together with the short-term design, these preliminary results should be interpreted cautiously, and larger, longer-term studies are warranted.

## Introduction

Badminton is one of the fastest racket sports, with shuttlecock velocities exceeding 300 km/h. In such high-intensity settings, athletes must rapidly process visual information and generate precise motor responses within milliseconds^1^. Consequently, visual performance—including binocular coordination, depth perception, and oculomotor control—constitutes a critical determinant of success on the court^2^. Beyond physical conditioning and technical proficiency, visual efficiency plays a pivotal role in supporting rapid perceptual–motor integration^3^. Among visual functions, vergence control—the coordinated inward and outward eye movements that sustain single binocular vision—is particularly crucial when gaze direction and object distance change abruptly. Athletes engaged in visually demanding sports often demonstrate superior vergence facility compared to non-athletes, suggesting that this ability is trainable and may contribute to performance enhancement^4,5^. Moreover, previous studies suggest that more efficient vergence responses may be associated with faster visuomotor reaction times, further linking binocular control to athletic outcomes.

Sports vision training (SVT) has gained recognition as a practical approach to enhance visual–motor capabilities, including tracking accuracy, stereopsis, and reaction time^6^. Accumulating evidence across multiple sports further supports the importance of vision in optimizing athletic performance. For example, professional and collegiate baseball players exhibit superior visual acuity, contrast sensitivity, and tracking skills compared to non-athletes, and these visual advantages correlate with batting performance^7,8^. In basketball, vision training has been associated with improvements in shooting accuracy, reaction time, and decision-making under pressure^9^. In soccer, gaze and perceptual–cognitive training have been shown to enhance scanning behaviors and passing accuracy^10^. Racket sports such as tennis and table tennis also rely heavily on anticipation and visuomotor coordination, both of which can be enhanced through targeted visual training^11^. Moreover, a recent systematic review concluded that sports vision training (SVT) not only reduces injury risk and improves agility but also facilitates adaptive motor behavior in both interceptive and strategic sports^5^.

Importantly, these findings indicate that SVT is beneficial not only in acute contexts but also in long-term developmental programs. A systematic review of 126 studies demonstrated that longitudinal SVT regimens—particularly those incorporating sport-specific stimuli and ecologically valid tasks—yield consistent improvements in both visual skills and athletic performance^11^. In parallel, immersive technologies such as virtual and augmented reality have been shown to provide safe, adaptable platforms for enhancing perceptual–cognitive load and decision-making in competitive scenarios^12^. Compared with such immersive technologies, prism-based approaches like automatic dual-rotational Risley prisms (ADRRPs) represent a more clinically accessible and standardized method to provide vergence-specific stimulation, making them suitable for both laboratory and applied sports settings^13,14^.

Among available SVT modalities, ADRRPs offer a controlled means of delivering dynamic vergence stimuli. Prior studies have demonstrated that ADRRPs can increase convergence amplitude, accommodative flexibility, and fusional range in non-athlete populations^13,14^. Nevertheless, despite encouraging evidence from extended training programs, the immediate effects of brief vergence training interventions on sport-related visual functions remain insufficiently investigated. Addressing this gap is essential, as even short interventions may be practical and valuable for athletes with constrained training schedules^15^.

Therefore, the present work is designed as an exploratory pilot study, aiming to provide preliminary evidence on whether a single 15-minute vergence training session with ADRRPs may influence binocular visual function and reaction time in youth badminton athletes. By simultaneously evaluating oculomotor and perceptual–motor outcomes, the study aims to clarify the feasibility and potential value of incorporating brief, targeted vision training into high-performance sports programs.

## Methods

### Participants

Twenty-six badminton athletes aged 18–25 from two academic institutions in Taiwan were recruited for this study. Ethical approval was granted by the Human Research Ethics Committee of the China Medical University Hospital, Taichung, Taiwan (CRREC-114-011). All participants provided written informed consent prior to data collection. All experimental procedures were performed in accordance with relevant guidelines and regulations, including the Declaration of Helsinki and institutional policies on human research ethics. Inclusion criteria were: (1) between 18 and 30 years old; (2) at least two years of continuous badminton training. Exclusion criteria were as follows: (1) a best-corrected visual acuity (BCVA) lower than 20/25 at distance or near in either eye; (2) presence of constant strabismus; (3) history of strabismus or refractive surgery; (4) diagnosed systemic conditions that may interfere with binocular vision (e.g., diabetes, multiple sclerosis); (5) history of neurological disorders or acquired brain injury.

All participants met the visual criteria, with BCVA of 20/25 or better in each eye, and refractive errors corrected using their habitual prescription (glasses or contact lenses as appropriate). All visual function assessments and training procedures were performed with refractive correction in place.

### Study procedure

Participants were randomly assigned to the visual training (VT) and control groups in an approximately 3:2 ratio due to limited participant availability using computer-generated randomization. A total of 16 participants (10 females, 6 males) were allocated to the VT group, and 10 participants (6 females, 4 males) to the control group.

Group allocation was masked from both participants and examiners and swas known only to the principal investigator. To ensure measurement reliability, all visual function parameters were consistently assessed by the same professor of optometry, while reaction time testing was conducted by the same professor of sports science.

The VT group underwent a single 15-minute session of vergence training using the ADRRPs (OrthoV Co., Ltd., Taiwan) system, which dynamically adjusted prism power based on each participant’s positive fusional vergence (PFV) break point to stimulate vergence responses. In contrast, the control group wore the same ADRRPs device equipped with plano lenses, which provided no vergence challenge. The rationale for this design was to establish a placebo condition and control for potential expectancy effects. Importantly, the device was motor-driven, producing the same operational sounds and vibrations as the training device but without prism-induced disparity.

**Fig. 1:**
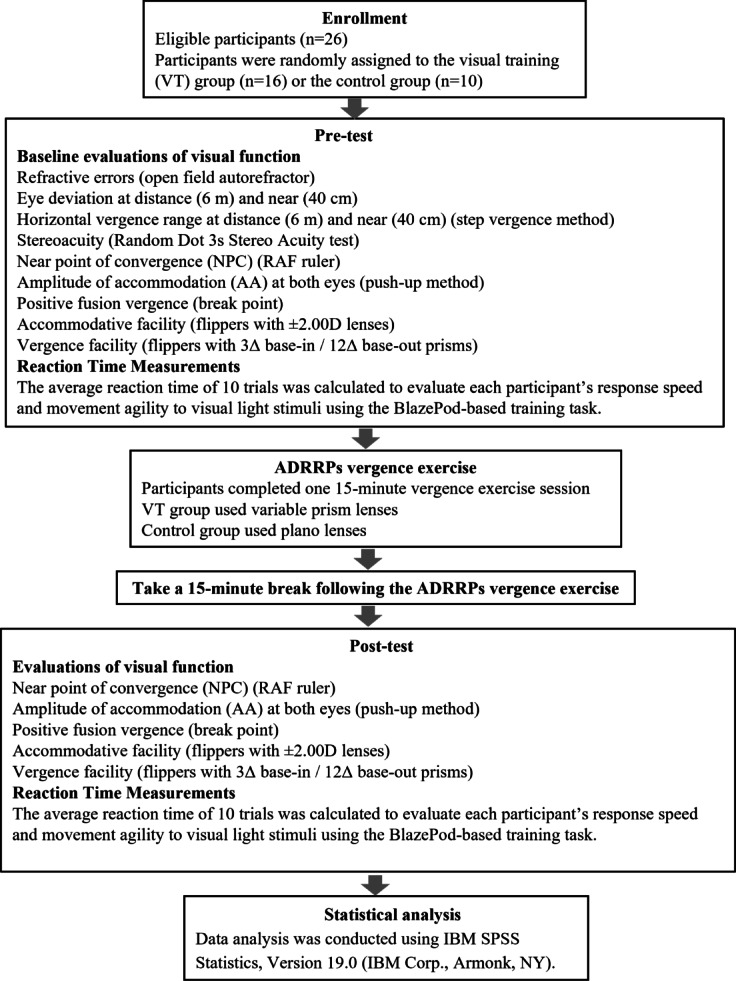
Flowchart of the experimental protocol. Visual function assessments and reaction time measurements were conducted at baseline and immediately after the visual training to evaluate the effects of ADRRPs-based intervention.

Visual function and reaction time were assessed at baseline and re-evaluated after a 15-minute rest period following the ADRRPs exercise. A visual overview of the study procedure is illustrated in Fig. 1.

### Visual function evaluation

Baseline assessments included visual acuity, refractive error, and stereoacuity to confirm normal binocular vision. Near point of convergence (NPC), amplitude of accommodation (AA), accommodative and vergence facility (AF, VF), and positive fusional vergence (PFV) were measured using standard clinical methods, and the obtained values were used for analysis. Negative fusional vergence (NFV) was not measured to minimize participant fatigue and testing time in the single-session design; instead, we prioritized PFV, AF, VF, AA, and NPC, which are more commonly used and clinically established indicators of vergence and accommodative adaptation in sports vision training.

### Vision screening

This study measured visual acuity (logMAR), refractive error (spherical equivalent), and stereoacuity (Random Dot Test) to confirm normal binocular visual status.

### Near point of convergence (NPC)

The participants’ NPC was assessed using a Royal Air Force (RAF) ruler. Participants tracked a 20/20 target as it was advanced toward their eyes at a rate of approximately 1–2 cm/s to ensure consistency across assessments. The break point, i.e., where diplopia occurred or one eye deviated, was recorded.

### Amplitude of accommodation (AA)

AA was assessed using the push-up method. Participants binocularly fixated on a near target that was advanced toward their eyes at a rate of approximately 1–2 cm/s until a blur was reported. The distance (in meters) was converted to diopters (D).

### Accommodative facility (AF) and vergence facility (VF)

We tested AF binocularly with ± 2.00 D flippers, and VF using 3∆ BI/12∆ BO prism flippers. Both were conducted at 40 cm with a 20/32 reduced Snellen target. The number of lens cycles cleared in one minute was recorded (cycles per minute, cpm). For both AF and VF, participants were instructed to report when the target was single and clear prior to each lens or prism flip. A separate suppression check was not performed because all participants demonstrated normal binocular vision and stereoacuity at baseline, making suppression unlikely.

### Positive fusional vergence (PFV)

PFV was measured using the step method at 40 cm with a phoropter. Base-out (BO) prisms were added until a break point occurred while maintaining fixation on a 20/20 near target, and the break point was recorded in prism diopters (∆).

### Reaction time task

**Fig. 2:**
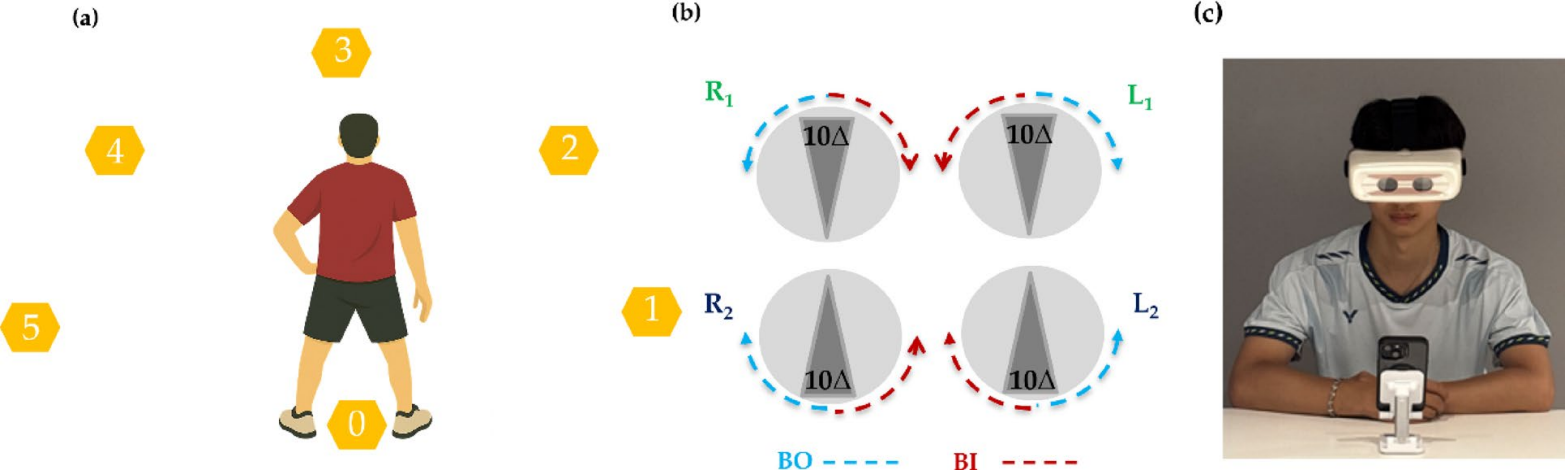
Illustrates the experimental setup. (a) Six BlazePod units were positioned in a semicircular arc with a 1.0-meter radius to assess reaction time. (b) The internal configuration of the Risley prisms within the ADRRPs device, along with a participant engaged in vergence training while viewing video content through the system.

Reaction time was evaluated using the BlazePod Trainer Kit, with six pods arranged in a semicircular configuration at a 1.0-meter radius, as shown in Fig. 2(a). Disc 0 (center) served as the starting point. Upon illumination of one of the outer discs (1–5), participants moved from Disc 0 to deactivate the light with their dominant hand, then re-turned to Disc 0. Ten randomized trials were performed. Average reaction time, recorded automatically, was the primary outcome.

### Vergence training protocol with ADRRPs

The ADRRPs device incorporated dual-rotating Risley prisms positioned in front of both eyes, allowing precise modulation of horizontal binocular disparity between base-in (BI) and base-out (BO) directions, with a maximum range of 40 prism diopters (20 prism diopters per eye) in either direction, as illustrated in Fig. 2(b). Prior to training, we measured each participant’s positive fusional vergence (PFV) break point to individualize the vergence stimulus. The BO setting was adjusted to exceed the PFV break point by + 2 prism diopters to provide a sufficient but tolerable convergence challenge, while the BI setting was set to approximately one-third of the BO value to balance divergence demand. All values were rounded to the nearest whole prism diopters. During each 10-second training cycle, the prism power increased toward the BO setting over one second. This was held for five seconds, shifted to the BI setting over one second, and held for an additional three seconds. The alternating sequence was repeated continuously for 15 min. Throughout training, participants wore the ADRRPs headset while viewing a video on a smartphone positioned at a distance of 40 cm. They were instructed to maintain clear and single binocular vision, as shown in Fig. 2(c).

### Statistics

Statistical analyses were conducted using IBM SPSS Statistics for Windows, Version 19.0 (IBM Corp., Armonk, NY, USA). Continuous variables were expressed as mean ± standard deviation (SD) for normally distributed data or as median with interquartile range (IQR) for non-normally distributed data. Normality was assessed prior to testing. Within-group comparisons between pre- and post-intervention measurements were performed using either paired-sample *t*-tests (for normally distributed data) or Wilcoxon signed-rank tests (for non-normal data), but not both. Between-group differences in change scores were evaluated using Welch’s *t*-tests. In addition, analysis of covariance (ANCOVA) was applied to examine group effects after adjusting for baseline values. Effect sizes (Cohen’s *d*) were calculated to quantify the magnitude of intervention effects. A *p*-value < 0.05 was considered statistically significant.

## Results

### Baseline

The baseline visual function data for the visual training (VT) group (*n* = 16) and the control group (*n* = 10) are summarized in Table 1. An independent samples t-test was conducted to compare baseline between experimental and control groups. Statistical comparisons indicate no significant differences between groups in refractive status, visual acuity, stereoacuity, or accommodative and vergence-related measures. The findings suggest that both groups were comparable at baseline before the intervention.

**Table 1:** Baseline measurements showed no significant differences between the control and vision training (VT) groups across all assessed parameters, including age, spherical equivalent (SE), best corrected visual acuity (BCVA), stereoacuity, accommodative amplitude (AA), accommodative facility (AF), near point of convergence (NPC), vergence facility (VF), and positive fusional vergence (PFV), confirming group equivalence prior to the intervention.

	VT	Control	*p*
N	16	10	
Age	19.5 ± 1.3	18.8 ± 1.1	0.200
OD SE (D)	−2.2 ± 1.8	−1.0 ± 1.2	0.083
OS SE (D)	−2.2 ± 1.8	−1.2 ± 1.3	0.113
OD BCVA (logMAR)	−0.1 ± 0.1	−0.1 ± 0.1	0.346
OS BCVA (logMAR)	−0.1 ± 0.1	−0.2 ± 0.1	0.052
Stereoacuity (sec)	26.3 ± 9.5	26.5 ± 10.6	0.968
AA (D)	10.9 ± 1.4	10.8 ± 1.2	0.551
AF (cpm)	18.5 ± 4.2	18.3 ± 3.7	0.645
NPC (cm)	6.90 ± 2.9	6.1 ± 1.1	0.341
VF (cpm)	20.9 ± 6.0	22.00 ± 4.1	0.601
PFV (△)	26.7 ± 7.9	22.4 ± 7.0	0.173

### Changes in vergence facility

At baseline, the mean VF was 20.9 ± 6.0 cpm in the training group and 22.0 ± 4.1 cpm in the control group, with no significant difference between them (*p* = 0.60). Following the intervention, the training group demonstrated a marked increase in VF (27.2 ± 7.1 cpm), whereas the control group showed only a slight change (23.7 ± 3.9 cpm).

Within-group analyses using paired t-tests revealed a significant improvement in the training group (paired t-test, *p* < 0.001), but not in the control group (paired t-test, *p* = 0.057). A between-group comparison of change scores confirmed that the training group improved significantly more than the control group (Welch’s t-test, *p* = 0.004). ANCOVA further indicated a significant group effect after adjusting for baseline (*p* < 0.01).

**Fig. 3:**
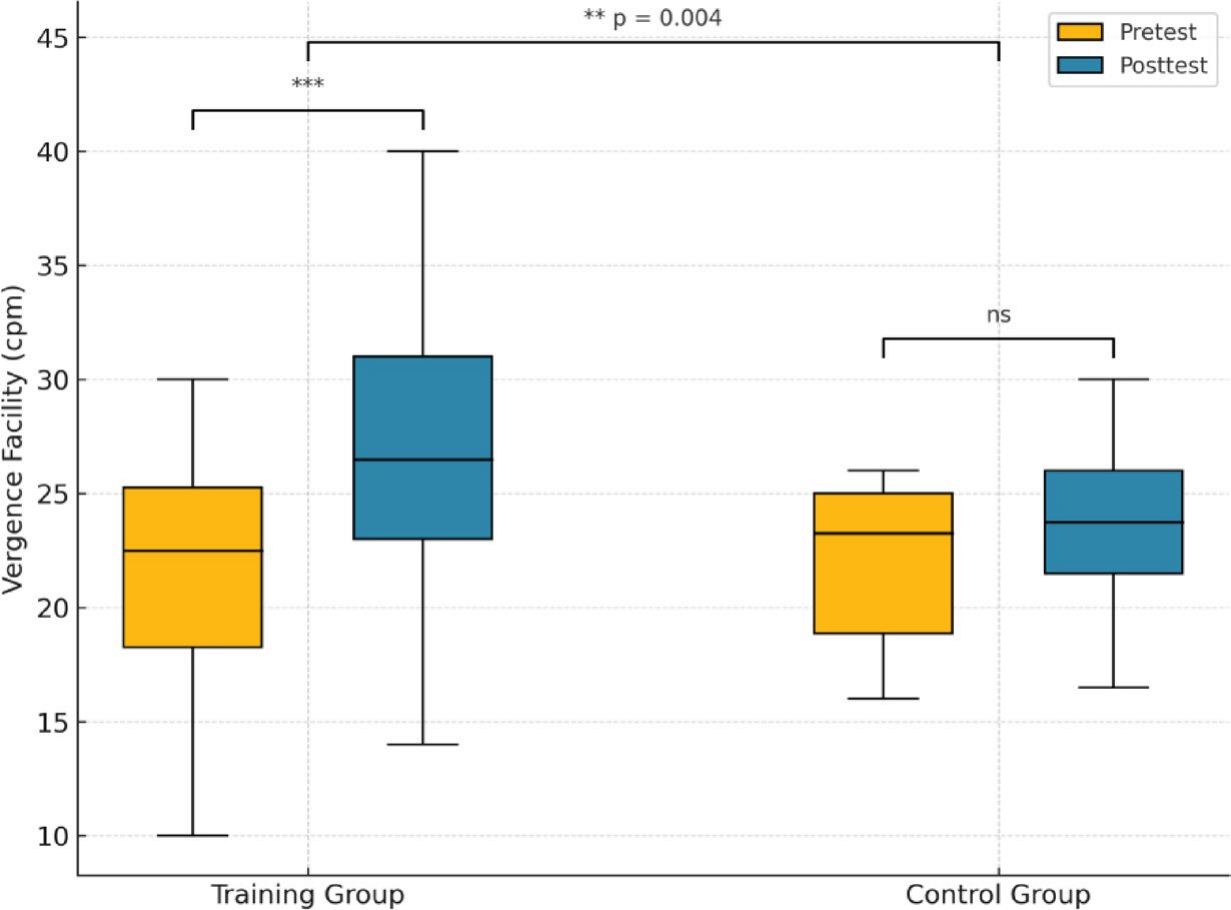
Boxplots of vergence facility (cycles per minute, cpm) at pretest (orange) and posttest (blue) in the visual training (VT) and control groups.

Figure 3 shows boxplots of vergence facility (cycles per minute, cpm) at pretest (orange) and posttest (blue) in the visual training (VT) and control groups. Each box represents the interquartile range (IQR), the horizontal line indicates the median, and whiskers extend to 1.5 × IQR. The VT group exhibited a significant improvement after a single ADRRPs-based vergence training session (***p < 0.001, paired t-test), whereas the control group showed no significant change (*ns*, *p* = 0.057). Between-group comparison of change scores confirmed that the improvement was significantly greater in the VT group than in the control group (**p = 0.004, Welch’s t-test). Statistical annotations are shown above the corresponding comparisons; however, results should be interpreted cautiously given the pilot nature of this study.

### Changes in positive fusional vergence break

At baseline, the mean PFV break was 26.7 ± 7.9 Δ in the training group and 22.4 ± 7.0 Δ in the control group, with no significant difference between them (*p* > 0.1). Following the intervention, the training group showed a slight increase in PFV break (28.5 ± 7.6 Δ), whereas the control group remained nearly unchanged (22.8 ± 7.9 Δ). Within-group analyses revealed no significant improvement in either group (training: paired t-test, *p* = 0.166; control: paired t-test, *p* = 0.662). A between-group comparison of change scores also showed no significant difference (Welch’s t-test, *p* = 0.459). ANCOVA further confirmed that there was no significant group effect after adjusting for baseline values.

### Changes in near point of convergence (NPC)

At baseline, the mean NPC was 6.9 ± 2.9 cm in the training group and 6.1 ± 1.1 cm in the control group, with no significant difference between them (*p* > 0.1). Following the intervention, the training group showed a slight reduction in NPC (6.2 ± 2.1 cm), whereas the control group also exhibited a small decrease (5.6 ± 1.0 cm). Within-group analyses revealed no significant improvement in either group (training: paired t-test, *p* = 0.151; control: paired t-test, *p* = 0.078). A between-group comparison of change scores showed no significant difference (Welch’s t-test, *p* = 0.589). ANCOVA further confirmed that there was no significant group effect after adjusting for baseline values.

### Changes in accommodative facility (AF)

At baseline, the mean AF was 18.5 ± 4.2 cpm in the training group and 18.3 ± 3.7 cpm in the control group, with no significant difference between them (*p* > 0.1). Following the intervention, the training group demonstrated a marked increase in AF (21.9 ± 4.6 cpm), whereas the control group showed only a slight, non-significant change (19.5 ± 4.0 cpm). Within-group analyses using paired t-tests revealed a significant improvement in the training group (paired t-test, *p* < 0.001), but not in the control group (paired t-test, *p* = 0.057). A between-group comparison of change scores confirmed that the training group improved significantly more than the control group (Welch’s t-test, *p* = 0.007). ANCOVA further indicated a significant group effect after adjusting for baseline (*p* < 0.01).

**Fig. 4:**
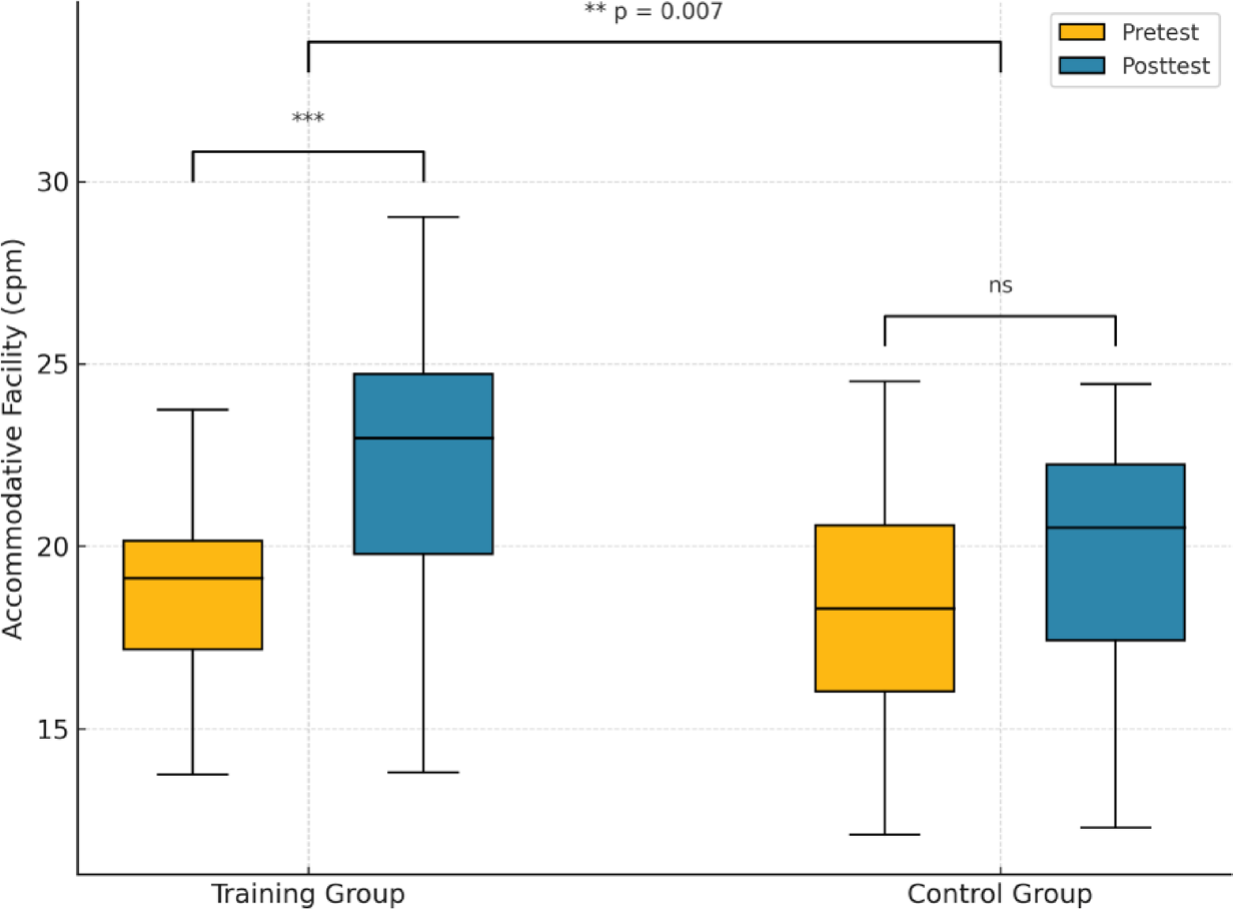
Boxplots of AF (cycles per minute, cpm) at pretest (orange) and posttest (blue) in the visual training (VT) and control groups.

Figure 4 shows boxplots of AF (cycles per minute, cpm) at pretest (orange) and posttest (blue) in the visual training (VT) and control groups. Each box represents the interquartile range (IQR), the horizontal line indicates the median, and whiskers extend to 1.5 × IQR. The VT group exhibited a significant improvement after a single ADRRPs-based vergence training session (***p < 0.001, paired t-test), whereas the control group showed no significant change (ns, *p* = 0.057). Between-group comparison of change scores confirmed that the improvement was significantly greater in the VT group than in the control group (**p = 0.007, Welch’s t-test). Statistical annotations are shown above the corresponding comparisons; however, results should be interpreted cautiously given the pilot nature of this study.

### Changes in amplitude of accommodation (AA)

At baseline, the mean AA was 10.9 ± 1.4 D in the training group and 10.8 ± 1.2 D in the control group, with no significant difference between them (*p* > 0.1). Following the intervention, the training group showed only a minimal change in AA (11.4 ± 3.5 D), whereas the control group demonstrated a slight, non-significant increase (11.0 ± 1.0 D). Within-group analyses revealed no significant improvement in either group (training: paired t-test, *p* = 0.082; control: paired t-test, *p* = 0.896). A between-group comparison of change scores also showed no significant difference (Welch’s t-test, *p* = 0.836). ANCOVA further confirmed that there was no significant group effect after adjusting for baseline values (*p* = 0.924).

### Changes in reaction time performance

At baseline, the mean reaction time was 1.14 ± 0.17 s in the training group and 1.09 ± 0.13 s in the control group, with no significant difference between them (*p* > 0.1). Following the intervention, the training group exhibited a significant reduction in reaction time (1.05 ± 0.13 s), indicating faster responses, whereas the control group showed no meaningful change (1.13 ± 0.13 s). The training group showed a significant reduction in reaction time (paired t-test, *p* = 0.001), whereas the control group did not (paired t-test, *p* = 0.055). A between-group comparison of change scores confirmed that the training group improved significantly more than the control group (Welch’s t-test, *p* < 0.001). ANCOVA further indicated a significant group effect after adjusting for baseline (*p* < 0.01).

**Fig. 5:**
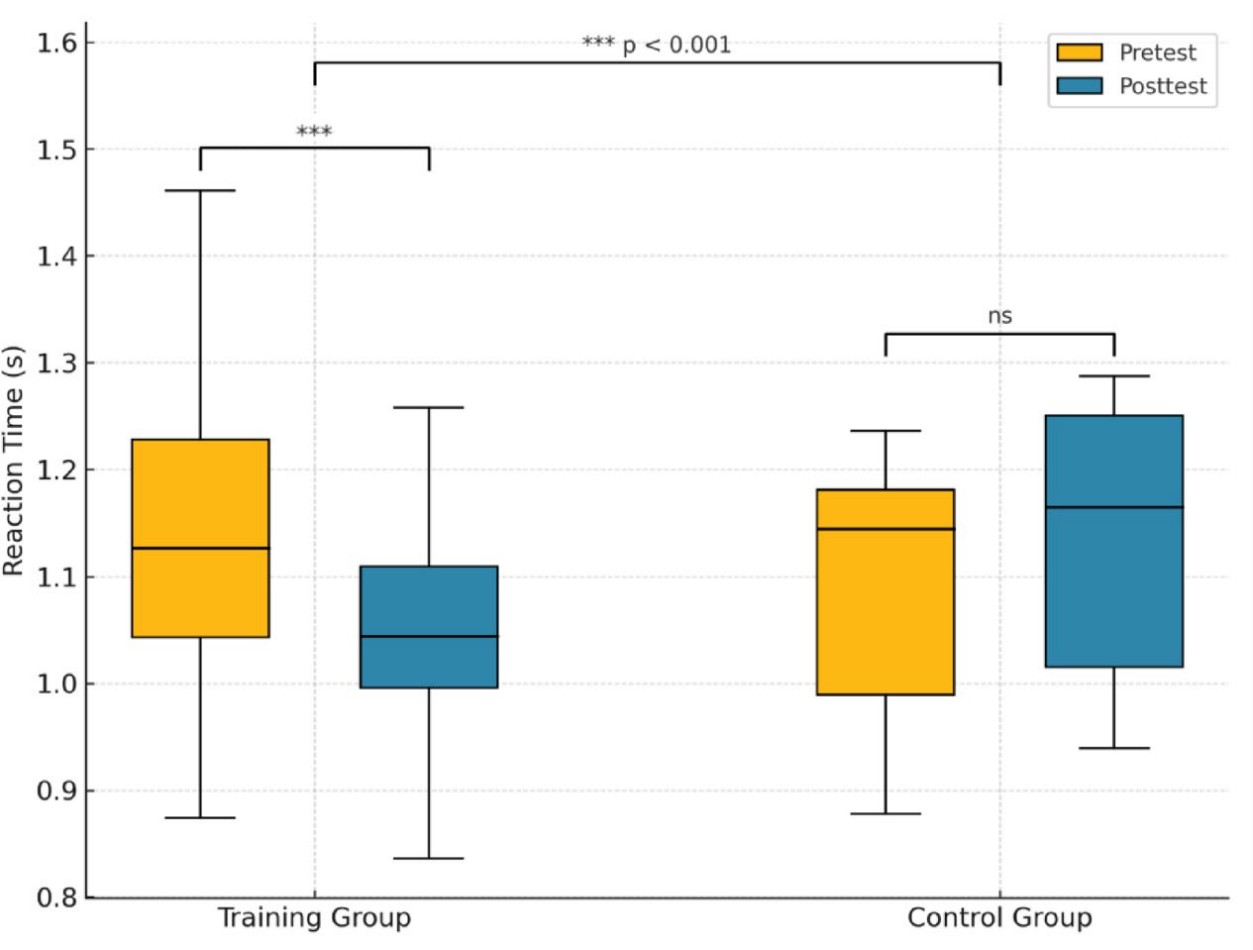
Boxplots of reaction time (s) at pretest (orange) and posttest (blue) in the visual training and control groups.

Figure 5 shows boxplots of reaction time (s) at pretest (orange) and posttest (blue) in the visual training and control groups. Each box represents the interquartile range (IQR), the horizontal line indicates the median, and whiskers extend to 1.5 × IQR. The VT group exhibited a significant improvement after a single ADRRPs-based training session (***p < 0.001, paired t-test), whereas the control group showed no significant change (ns, *p* = 0.055). Between-group comparison confirmed that the reaction time improvement was significantly greater in the VT group than in the control group (***p < 0.001, Welch’s t-test). Statistical annotations are shown above the corresponding comparisons; however, results should be interpreted cautiously given the pilot nature of this study.

## Discussion

This study examined the immediate effects of a single-session vergence training protocol utilizing automatic dual-rotational Risley prisms (ADRRPs) on visual function and reaction time in youth badminton athletes. The findings indicate that even a brief, one-time intervention may enhance specific oculomotor abilities and visuomotor responsiveness.

Contrary to past research primarily focused on extended or multi-week visual training regimens, our results suggest that targeted, short-duration interventions may provide preliminary benefits. We observed significant improvements in vergence facility (VF) and accommodative facility (AF), which exhibited moderate to large effect sizes. These outcomes suggest the adaptability of the oculomotor system to acute vergence stimuli. Similarly, Erickson (2021) highlighted that vergence and accommodative facility are critical visual functions in sports that demand rapid shifts in focus, further supporting the relevance of our findings^16^.

The observed reduction in reaction time reinforces the potential benefits of brief vergence interventions. This result is consistent with prior evidence that vision training can enhance visuomotor processing and reaction efficiency in athletes^17^. While the improvement in the VT group may reflect greater visuomotor efficiency from vergence training, a learning effect from repeated testing cannot be entirely excluded. However, the slight, non-significant increase observed in the control group suggests that practice effects alone are unlikely to account for the gains. Supporting evidence comes from studies in other sports: collegiate baseball players who underwent structured vision training demonstrated not only improved stereopsis but also higher batting averages compared to untrained peers^4^. Similarly, vision-based interventions have been linked to faster reaction times and better decision-making in soccer, basketball, and hockey players^18^.

In line with Pokaisasawan et al., who reported immediate improvements in AF and amplitude of accommodation (AA) following manual pencil push-up exercises, our results suggest that device-based training via ADRRPs may elicit rapid visual gains^15^. However, the ADRRPs system offers a more automated, standardized approach, which may be better suited for structured sports settings. Compared with immersive VR/AR systems, ADRRPs may provide a more accessible, standardized, and clinically applicable approach to vergence-specific training.

Beyond performance enhancement, vision training has also been linked to injury prevention. Studies indicate that preseason visual conditioning can reduce concussion incidence in collegiate football and soccer by improving athletes’ visual awareness and reaction to external stimuli^16^. Although our study did not directly assess injury outcomes, such evidence suggests broader applications of vergence-based protocols.

Despite these promising outcomes, only minimal changes were observed in AA, with a negligible effect size in the training group. This finding may suggest that accommodation is less responsive to short-term vergence-based stimuli or may require distinct protocols to achieve noticeable improvement. Moreover, changes in near point of convergence (NPC) and positive fusional vergence (PFV) did not reach statistical significance. These results align with prior reports that certain oculomotor functions benefit more from repeated exposure or multimodal training approaches^19^.

These preliminary findings may indicate the potential feasibility of ADRRPs-based vergence training as an exploratory method for enhancing essential visual functions and reaction speed in athletes. Future studies should expand the range of vergence parameters, include long-term follow-up, and explore cumulative or multimodal training effects to determine their impact on sport-specific performance, particularly in badminton and other fast-paced sports.

### Limitations

This study has several limitations. First, there was no objective monitoring of fusion during training, so we cannot confirm that participants maintained single binocular vision throughout the ADRRPs session. The small sample size and unequal group allocation may limit statistical power and generalizability. Moreover, only the immediate effects of a single session were examined, so the durability of improvements remains unknown. Sport-specific outcomes such as on-court accuracy or agility were also not assessed, restricting direct translation to athletic performance. In addition, the vergence parameters measured in this study were limited to near vision assessments, whereas athletes typically rely on distance vision during actual gameplay. This restricts the generalizability of our findings to sport-specific visual demands. Future studies should therefore include distance vergence measures and examine correlations between changes in reaction time and vergence parameters to better understand their relationship.

Finally, not all vergence parameters were measured, and improvements in reaction time could partly reflect learning effects, though the control group’s trend suggests otherwise. These results may not generalize to other sports or age groups. As a pilot study with a sport-specific cohort, these findings should be viewed as preliminary, and future research with larger samples, broader measures, and long-term follow-up is needed.

## Conclusions

This exploratory pilot study suggests that a brief, single-session vergence training that a brief, single-session vergence training with automatic dual-rotational Risley prisms (ADRRPs) may produce immediate, measurable enhancements in key oculomotor functions and visuomotor responsiveness among youth badminton athletes. Significant gains were observed in vergence facility and accommodative facility, together with a marked reduction in reaction time, underscoring the adaptability of the visual system to short-duration, targeted interventions. However, it remains unknown how long these improvements are maintained.

Other visual measures, including amplitude of accommodation, near point of convergence, and positive fusional vergence, showed only minimal or non-significant changes, suggesting that certain oculomotor functions may require repeated or longer training protocols to elicit meaningful improvements. These findings may indicate the potential feasibility of ADRRPs-based vergence training as a practical and efficient tool for sports vision enhancement.

Future research should investigate the cumulative effects of repeated sessions, incorporate long-term follow-up, and directly assess sport-specific performance outcomes to clarify the broader value of vergence training in competitive settings.

## Data Availability

The datasets from this study are available from the corresponding author upon request.

## Abbreviations

ADRRPs: Automatic Dual-Rotational Risley prisms
VT: Visual Training
VF: Vergence Facility
AF: Accommodative Facility
AA: Amplitude of Accommodation
NPC: Near Point of Convergence

## Declarations

### Patents

Two Taiwan invention patents (I781072 and I878069) have been granted for the automatic dual rotational Risley prisms system.
